# Pharmacogenomics: From classroom to practice

**DOI:** 10.1002/mgg3.417

**Published:** 2018-05-31

**Authors:** Samantha C. Nutter, Marina Gálvez‐Peralta

**Affiliations:** ^1^ Department of Pharmaceutical Sciences West Virginia University School of Pharmacy Morgantown West Virginia

## Abstract

Perceptions and challenges connecting Pharmacogenomics taught in classrooms and translationing it to advance pharmacy practice rotations and healthcare settings and potential areas of development.

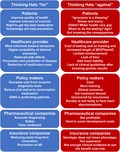

## 1 PHARMACOGENOMICS EDUCATION IN THE UNITED STATES

Lucretius was one of the pioneers on the idea of personalized medication, suggesting that patients could respond to the same treatment differently (Lucretius Caro, 65 B.C.). Vogel introduced the term “pharmacogenetics” in the early 50s, explaining how different genetic background could predispose some patients to a different outcome after their medication was administered, causing either toxicity or therapeutic failure (Vogel, 1959). Other examples that followed Vogel's variability on drug response were the discoveries of primaquine‐induced hemolytic anemia (Beutler, 1993), succinylcholine‐induced apnea (Kalow, 1964), or isoniazid‐induced hepatotoxicity (Blum, Demierre, Grant, Heim, & Meyer, 1991).

Pharmacists are the expert on the medication, and with that in mind, the American Board of Colleges of Pharmacy (ABCP), incorporated Pharmacogenomics’ education as a requirement in their Accreditation Council for Pharmacy Education (ACPE) Standards in 2015, requesting that pharmacy students should be prepared to understand and apply Pharmacogenomics concepts to patient care (Accreditation Council for Pharmacy Education, 2015).

Moreover, other health organizations such as the National Coalition for Health Professional Education in Genetics (NCHPEG; Johnson et al., 2002; Kenner, 1998; Mclnerney, 2007), the International Society of Pharmacogenomics (Gurwitz et al., 2005), the American College of Clinical Pharmacy (ACCP; American College of Clinical Pharmacy, 2000), the American Society of Hospital Pharmacists (ASHP; Feero, Kuo, Jenkins, & Rackover, 2012; Haidar, Hoffman, & Johnson, 2015), and the NIH‐funded Genetic/Genomics Competency Center (G2C2; Accreditation Council for Pharmacy Education, 2015), have joined efforts to propose recommendations on how to either educate, or implement pharmacogenomics in the classrooms and in the clinic. These organizations stated that: “Pharmacist's responsibilities are to educate patients about pharmacogenomic principles and advocate for the rational and routine use of pharmacogenomic testing in appropriate indications” (Haidar et al., 2015).

The most recent literature describing scholarship on education or healthcare interventions reflects this trend describing different examples of how pharmacogenomics can be incorporated in the PharmD program (Feero & Green, 2011; Formea et al., 2013; Frueh, Goodsaid, Rudman, Huang, & Lesko, 2005; Frueh & Gurwitz, 2004; Gurwitz et al., 2005; Harirforoosh et al., 2009).

Pharmacy Schools are incorporating a wide variety of educational approaches and strategies to teach pharmacogenomics, which include didactic lectures (Brazeau & Brazeau, 2006), clinical exercises and online resources (Farrell, Goodbar, et al., 2014, Galvez‐Peralta, Szklarz, Geldenhuys, & Lockman, 2017), personal genomic sequencing (Adams et al., 2016; Knoell, Johnston, Bao, & Kelley, 2009; Krynetskiy & Calligaro, 2009; Nickola & Munson, 2014), laboratory exercises (Farrell, Pedigo, & Messersmith, 2014), ‐ bioinformatics (Springer, Iannotti, Kane, Haynes, & Sprague, 2011), experiential activities (Drozda et al., 2013; Lee et al., 2012) among others.

The integration of Pharmacogenomic education is being successful in PharmD programs. While in 2005 only 32 out of 41 (78%) schools of Pharmacy that answered a survey described that they have some components of pharmacogenomics in their curriculum (Latif & McKay, 2005), in 2010, the number increased to 69 out of 75 (92%; Murphy et al., 2010). However, 40% of these schools were offering 10 or less didactic hours in pharmacogenomics (Murphy et al., 2010), and in some situations just as an elective.

## 2 HEALTHCARE PROVIDERS’ PERCEPTION OF PHARMACOGENOMICS

Although pharmacogenomics education has been introduced over the past few years in pharmacy programs, practicing pharmacists’ confidence is not growing at parallel pace (de Denus et al., 2013; Haidar et al., 2015; Johnson et al., 2002; Mccullough et al., 2011; Sansgiry & Kulkarni, 2003; Tuteja et al., 2013). Even worse, physicians and other healthcare providers have revealed a lack of confidence or awareness to discuss pharmacogenomic information, testing, or educating patients (Brierley et al., 2012; Haga, Burke, Ginsburg, Mills, & Agans, 2012; Kelly et al., 2012; Passamani, 2013; Stanek et al., 2012). Some studies report that just 10%–13% of physicians who participated in the study felt confident with their knowledge (Haga et al., 2012; Stanek et al., 2012).

## 3 BRIDGING THE GAP

With these challenges in mind, different activities were created in a first‐year course on pharmacogenomics for pharmacy students at our School of Pharmacy. For two consecutive years, a debate on personalized medicine was generated, with modifications. In the first year, a classroom debate was organized, in which all 76 students from the class were divided into groups, and randomly assigned them as “for” or “against” personalized medicine. The three biggest issues that the “against” groups presented were cost and training, discrimination, and potential risk of generating anxiety to patients and their family members after discovering a risk to develop an untreatable disease. A survey was offered to the class after completion of the experience, and results shown that some students changed their personal perspective based on the debate. (Galvez‐Peralta et al., 2017).

**Figure 1:**
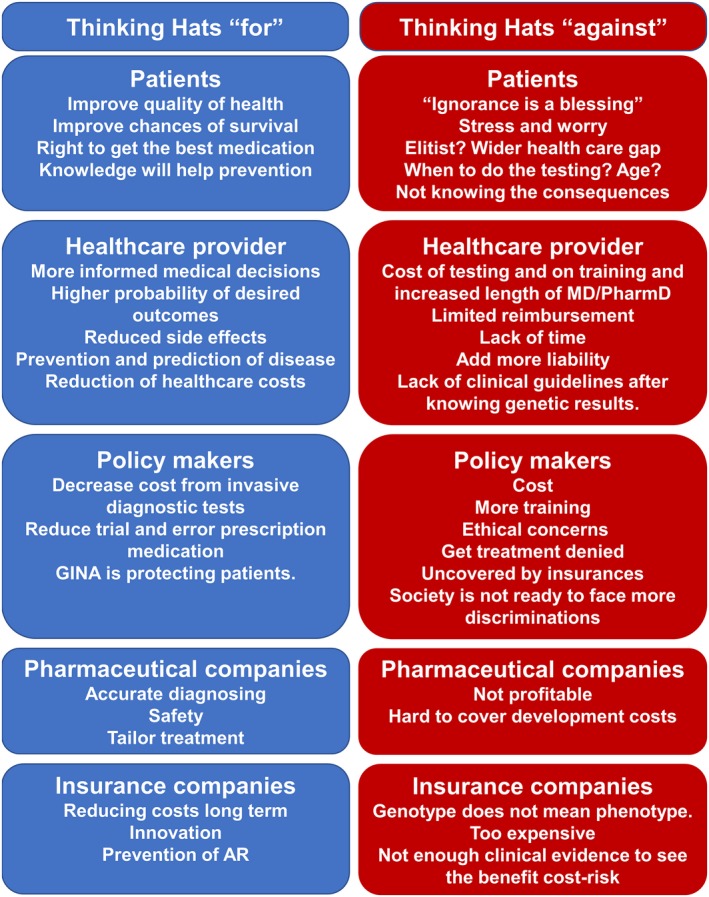
P1 2017 Students’ statements presented in an online debate using the “thinking‐hat” approach: In blue (light gray in print): Patients, healthcare providers, insurance companies, policy makers ,and pharmaceutical companies that are “for” pharmacogenomics. In red (dark gray in print): Patients, healthcare providers, insurance companies, policy makers, and pharmaceutical companies that are “against” pharmacogenomics. GINA (Genetic Information Non‐discrimination Act); AR (Adverse reaction)

In the following year, few modifications were offered to the class: first, the debate was asynchronous, using an online platform where students could post their perceptions, to mimic the current Millennials’ communication tendencies; second, rather than dividing the teams into “for” or “against,” a “thinking‐hat” approach was used. The “thinking‐hat” consists of asking students to put themselves under a particular situation. In this case, the different hats were: “patients for” or “against”; “healthcare provider for” or “against”; “insurance companies for” or “against”; “policy makers for” or “against”; and “pharmaceutical companies for” or “against” pharmacogenomics/personalized medicine. The main conclusions presented by the different teams are summarized in Figure 1. Rather than offering a survey at the end, students were asked to submit a self‐reflection of their personal point of view after the debate was closed, to gain more qualitative data and to learn how the debate and thinking under different aspects could have influenced each student's perception. Interestingly, out of the 67 students that were part of the debate, only four of them thought that the challenges and negative impact of pharmacogenomics could outweigh the positive aspects that pharmacogenomics could bring. Two students were on the fence, not being able to decide if they were for or against.

Some of the students’ reflections discussed some barriers for implementation, such as liability, cost, who will cover the cost, consequences of errors (i.e., providing the wrong genetic test result), privacy, affordability, discrimination, lack of time from the healthcare provider, or potential future research revealing potential risk of particular “pharmacogenes” involved in risks for specific diseases.

Overall, after additional exercises in the course, like getting familiarized with CPIC and PharmGKB, 91% of students shared that they were confident or very confident on discussing pharmacogenomics with patients or healthcare providers (Galvez‐Peralta et al., 2017). These results are similar to other schools that have described how new pharmacy students understand the significance of pharmacogenetics and pharmacogenomics for their future as pharmacists (Lee, Hudmon, Ma, & Kuo, 2015) and the importance to improve patient care (Moen & Lamba, 2012).

There is evidence that supports a deep commitment made by pharmacy educators to provide pharmacogenomics education among all health sciences disciplines. Pharmacists are expected to advise clinicians on matters related to implementation of pharmacogenomics in patient care (Mccullough et al., 2011; Rao, Mayhew, & Rao, 2015), and to ensure the use of pharmacogenomics testing when appropriate for medication therapy (Haidar et al., 2015; Swen et al., 2008). Furthermore, some pharmacists are also involved in developing prescribing guidelines such as those from the Clinical Pharmacogenetics Implementation Consortium (CPIC; Daly, 2014), as well as in the application of pharmacogenomics principles in practice (El‐Ibiary, Cheng, & Alldredge, 2008; Ferreri et al., 2014; Owen, Director, & Practice, 2011; Padgett, O'Connor, Roederer, McLeod, & Ferreri, 2014; Weitzel et al. 2016). Despite all the efforts dedicated in the classrooms, the current reality is a little behind of what the students are being trained for. The hope is that, thanks to the new education, these well‐trained students will become advocates of pharmacogenomics on the profession and the healthcare system.

## 4 TRANSITION FROM CLASSROOM TO ADVANCED PHARMACY PRACTICE EXPERIENCE

As educators caring for how students will implement the foundational knowledge learned in classrooms, we need to pay attention to the reality that our students are exposed to when attending advanced pharmacy practice experience rotations (APPE) or after graduation. Ms. Nutter, an APPE student, shared her experience during her rotations in community pharmacy settings, with promising and discouraging experiences. These are some of her perceptions and experiences:

“As an Advanced Pharmacy Practice Experience (APPE) student, the biggest setbacks to pharmacogenomics is how to implement and use this new discipline in our daily practices and what are the potential ethical repercussions of doing these pharmacogenomic tests. There are some obvious uses for pharmacogenomics, such as genotyping in oncology, however, pharmacogenomics is much more diverse and could be used in other settings.

The American Society for Health‐Systems Pharmacy (ASHP) believes pharmacists have an important role in the clinical application of pharmacogenomics. They encourage education in the pharmacy curriculum and even recommend specialized training certificates for those interested. ASHP states “all pharmacists should have a basic understanding of pharmacogenomics in order to provide patient care that incorporates pharmacogenomics recommendations” (Feero et al., 2012). As I am now a fourth‐year pharmacy student, I had the chance to work in a variety of settings with a variety of pharmacists. Through my experiences, I believe most of what ASHP is saying is starting to surface in everyday practice. Pharmacists in West Virginia do possess a basic understanding of pharmacogenomics, and for those that did not have the pharmacogenomics training when they were in the program, the gap can be filled through continuous education courses. I think the issue lies in the second half of what ASHP stated‐ that Pharmacists should be able to *incorporate* that basic knowledge of pharmacogenomics in their daily practice (Haidar et al. 2015). Here is where I have seen a major deficiency during my rotations, and would like to share two examples:

While on a rotation at a community pharmacy, a patient came in complaining of sertraline toxicities after taking it for just a few weeks and dose de‐escalation (i.e., reducing initial dosing that was prescribed due to side effects). She stated that her sister had been taking the same medication for years with no issues and her sister was the one suggesting her to ask our patient's physician for this particular medication. My preceptor counseled the patient making sure that she was taking the medication properly, as prescribed. Furthermore, he reviewed the patient's medical record to assure that there were no potential drug–drug interactions with other medications that the patient was taking concurrently. After discarding these two possibilities, my preceptor explained how everyone could respond differently to the same medication. My preceptor counseled and suggested to the patient to ask for an alternative medication to her doctor. After he finished I mentioned the possibility of genetic testing. I explained, after hearing the issues the patient was having, that she could potentially be a poor metabolizer for sertraline and the drug could be building in her body or a variant on the serotonin receptor, and that could be the cause for the problems that she was describing. The patient and my preceptor were both receptive to my suggestion. After the patient left, my preceptor thanked me, since he would have never considered that possibility. The patient was unfortunately lost to be followed up after my rotation and sadly, I am not aware if she got genetic testing or not. I think this is a prime example of practicing pharmacists understanding the value of pharmacogenomics but are still unsure of where it fits into their practice, or when to make certain recommendations to patients.

Not all my encounters with my preceptors went as smoothly as the previous example. On a different rotation at a community pharmacy settings, much of the prescriptions came from the physician's office next door. The physician prescribed clopidogrel for every patient that needed an antiplatelet medication (i.e., “blood thinners” as our patients call them). My rotation lasted 5 weeks and I did not see the physician write a prescription for any other antiplatelet other than clopidogrel. This was concerning to me because, as we know, this medication is a prodrug and needs to be metabolized through CYP2C19 to generate the active compound that will block the platelet aggregation. The FDA label includes a warning on this matter, indicating that patients who are poor metabolizers may not respond to this medication, and suggest using a different medication that is not metabolized through the same pathway (FDA, 2010). Since genotyping is not currently required for all patients taking clopidogrel, I suggested to my preceptor that we could counsel new patients about the option of genetic testing. He did not care for the idea, thinking it could cause more problems or liability issue for the pharmacy, and assuming that the physician was probably taking care of that type of information. This interaction with my preceptor solidified my passion for pharmacogenomics and reassured my perception that we need more advocates for pharmacogenomics testing in the patient care settings.

I have been interested in pharmacogenomics since my undergraduate courses and now I want to pursue a career in this field of pharmacy. I attended ASHP's Midyear convention and sought out a PGY‐1 residency program that offered pharmacogenomics is their curriculum. I met many of these programs’ directors at the convention to find out more about what their courses entailed. Pharmacogenomics courses and/or research on pharmacogenomics are being established in some PharmD residency programs. This, to me, is very exciting and promising to further pharmacogenomics in different diseases. However, I did run into some programs advertising pharmacogenomics as either just electives or courses that were still not functioning.

Right now, there are only a limited number of PharmD residency programs offering pharmacogenomics. The hope is that through, ASHP, and its goal of promoting pharmacogenomics in PharmD residency programs, these start to take notice”.

## 5 CONCLUSION

There is no doubt that the education of pharmacogenomics in Schools of Pharmacy across the country is flourishing, providing high‐quality and well‐prepared pharmacists, becoming leaders of pharmacogenomics. But, all this effort could be wasted if there is not an investment from the rest of the society and health care providers: By training practicing pharmacists and physicians, so the new graduating pharmacists could suggest genotyping patients during the monitoring treatment medication procedures (MTM), and not have the frustration of being a science that is only possible in the books or in science fiction; by investing in further research to have better recommendations and guidelines for each particular genotype; by designing better policies to protect patients; and finally, by proving that the cost is an investment for insurance companies, and patients will not be the ones suffering the burden of cost.

There is hope that the health care system could move forward, same as schools of pharmacy and their education already did. It can be possible.

## CONFLICT OF INTERESTS

None declared. WVU Office of Research Integrity and Compliance approved protocol: 1612373673.
